# VIPR: A probabilistic algorithm for analysis of microbial detection microarrays

**DOI:** 10.1186/1471-2105-11-384

**Published:** 2010-07-20

**Authors:** Adam F Allred, Guang Wu, Tuya Wulan, Kael F Fischer, Michael R Holbrook, Robert B Tesh, David Wang

**Affiliations:** 1 Departments of Molecular Microbiology and Pathology & Immunology, Washington University School of Medicine, St. Louis, Missouri USA; 2 Department of Pathology, University of Utah School of Medicine, Salt Lake City, Utah USA; 3 Department of Pathology, University of Texas Medical Branch, Galveston, Texas USA; 4 NIH Integrated Research Facility, Division of Clinical Medicine, 8200 Research Plaza, Fort Detrick, Frederick, MD 21702

## Abstract

**Background:**

All infectious disease oriented clinical diagnostic assays in use today focus on detecting the presence of a single, well defined target agent or a set of agents. In recent years, microarray-based diagnostics have been developed that greatly facilitate the highly parallel detection of multiple microbes that may be present in a given clinical specimen. While several algorithms have been described for interpretation of diagnostic microarrays, none of the existing approaches is capable of incorporating training data generated from positive control samples to improve performance.

**Results:**

To specifically address this issue we have developed a novel interpretive algorithm, VIPR (**V**iral **I**dentification using a **PR**obabilistic algorithm), which uses Bayesian inference to capitalize on empirical training data to optimize detection sensitivity. To illustrate this approach, we have focused on the detection of viruses that cause hemorrhagic fever (HF) using a custom HF-virus microarray. VIPR was used to analyze 110 empirical microarray hybridizations generated from 33 distinct virus species. An accuracy of 94% was achieved as measured by leave-one-out cross validation. *Conclusions*

VIPR outperformed previously described algorithms for this dataset. The VIPR algorithm has potential to be broadly applicable to clinical diagnostic settings, wherein positive controls are typically readily available for generation of training data.

## Background

The field of viral diagnostics, which has traditionally followed a "one virus-one assay" paradigm, has been revolutionized by the introduction of diagnostic microarrays [1-10]. It is now possible to test for the presence of thousands of viruses simultaneously in a single assay. A microarray-based approach is particularly effective for viral diagnosis of diseases that have a common phenotype, but may be caused by any of a number of different viruses. For example, acute respiratory disease, encephalitis and hemorrhagic fever are all disease syndromes known to be caused by a spectrum of viral pathogens. Microarrays specifically focused on the diagnosis of respiratory disease [11-14] and encephalitis [3-5] have been described, as have much broader pan-viral microarrays [1,2,8]. A wide range of probe design strategies and microarray platforms can be used for diagnostic microarrays. Independent of the probe design strategy or platform, a key component that is absolutely essential for all diagnostic microarrays is an objective method for interpreting the raw hybridization patterns. While many diagnostic microarrays have been described, there are only three published algorithms, E-Predict [15], DetectiV [16] and PhyloDetect [17], with downloadable or web-accessible software that are available for analyzing data from diagnostic microarrays.

The typical goal of diagnostic virology assays is to determine the presence or absence of one or more viruses from a finite, defined list of candidate viruses known to cause the disease in question. In clinical laboratories, samples of each candidate virus to be detected are typically readily available and can be used as positive controls. Our goal was to develop an interpretive algorithm for diagnostic microarrays that could take advantage of the existence of such positive controls to generate a training data set to guide subsequent analyses.

Toward this end, we developed a probabilistic algorithm for the purpose of analyzing diagnostic microarrays. This class of algorithms has been applied to numerous problems in biology. For example, hidden Markov models (HMMs) and the more youthful conditional random fields (CRFs) have allowed researchers to make important inferences about sequence structure and function [18,19]. Bayesian inference in a probabilistic framework offers a distinct advantage of capitalizing on empirical data to guide future predictions as compared with methods that are based solely on computational prediction of genome-to-probe binding. In addition, the power of utilizing probabilities as opposed to discretizing a host of parameters when considering possible solutions means that global calculations are less likely to be influenced by poor choices made locally. To date, no Bayesian algorithm for diagnostic microarrays has been described.

In this paper, we describe a novel probabilistic algorithm that relies on Bayesian inference for analysis of diagnostic microarrays. To validate this approach, we focused on analysis of the set of viruses known to cause hemorrhagic fever. HF symptoms include severe vascular damage, hemorrhage, high fever, and shock and can frequently lead to death [20,21]. HF viruses belong to four virus families: *Arenaviridae*, *Bunyaviridae*, *Flaviviridae *and *Filoviridae*. A custom microarray was designed to detect all known HF viruses and many of their close relatives. Specimens representing virtually every virus species known to cause HF were procured and hybridized to microarrays for the purpose of validating our algorithm. Furthermore, we compared VIPR's performance to that of the existing interpretive algorithms that are not capable of utilizing training data in this fashion.

## Methods

### Microarray design

14,864 oligonucleotide probes were designed using a taxonomy-based approach as described previously [22] except that the Agilent^® ^8 × 15 K platform was used and probes were 35, 45 or 60 nucleotides in length. The probes were designed to bind to viral genomes from the four families that contain all viruses known to cause HF: *Arenaviridae*, *Bunyaviridae*, *Filoviridae*, and *Flaviviridae*. Probes of different lengths were designed to account for different levels of conservation between viral taxa. For example, longer probes were included to represent regions of strong conservation, while shorter probes were included to distinguish closely related virus species in order to increase specificity.

### Hybridization of HF viruses to microarray

A total of 51 strains of 33 distinct virus species (see Table 1) obtained from the World Reference Center for Emerging Viruses and Arboviruses were grown in either Vero cells or C6/36 cells. RNA was extracted using standard Trizol^® ^protocols and was randomly amplified as previously described [2]. The resulting amplified material was then coupled to a fluorescent dye and hybridized to the HF microarray. Raw data measurements were collected using GenePix Pro^® ^software. In total, 110 hybridizations were performed (102 positive controls + 4 Vero negative controls + 4 C6/36 negative controls). All raw microarray data are available in NCBI GEO (accession GSM534862 through GSM534971). These 110 hybridizations constituted a set of positive and negative controls used for validation, a subset of which was used in training our algorithm.

**Table 1:** Viruses hybridized to the diagnostic microarray

Virus	Family	Causes HF	# of strains hybridized
Amapari virus	*Arenaviridae*	No	1

Guanarito virus	*Arenaviridae*	Yes	4

Ippy virus	*Arenaviridae*	No	1

Junin virus	*Arenaviridae*	Yes	1

Lassa virus	*Arenaviridae*	Yes	2

Lymphocytic choriomeningitis virus	*Arenaviridae*	No	1

Machupo virus	*Arenaviridae*	Yes	1

Mobala virus	*Arenaviridae*	No	1

Mopeia virus	*Arenaviridae*	No	1

Sabia virus	*Arenaviridae*	Yes	1

Tacaribe virus	*Arenaviridae*	No	1

California encephalitis virus	*Bunyaviridae*	No	1

Crimean-Congo hemorrhagic fever virus	*Bunyaviridae*	Yes	4

Hantaan virus	*Bunyaviridae*	Yes	1

La Crosse virus	*Bunyaviridae*	No	1

Ngari virus	*Bunyaviridae*	Yes	1

Puumala virus	*Bunyaviridae*	Yes	1

Rift Valley fever virus	*Bunyaviridae*	Yes	3

Seoul virus	*Bunyaviridae*	Yes	1

Toscana virus	*Bunyaviridae*	No	1

Angola marburgvirus	*Filoviridae*	Yes	1

Reston ebolavirus	*Filoviridae*	No	1

Sudan ebolavirus	*Filoviridae*	Yes	1

Zaire ebolavirus	*Filoviridae*	Yes	1

Gabon ebolavirus	*Filoviridae*	Yes	1

Dengue virus 1	*Flaviviridae*	Yes	2

Dengue virus 2	*Flaviviridae*	Yes	2

Dengue virus 3	*Flaviviridae*	Yes	2

Dengue virus 4	*Flaviviridae*	Yes	2

Kyasanur Forest disease virus	*Flaviviridae*	Yes	2

Omsk hemorrhagic fever virus	*Flaviviridae*	Yes	4

Rocio virus	*Flaviviridae*	No	1

Yellow fever virus	*Flaviviridae*	Yes	2

### VIPR normalization and transformation (Figure 1A)

**Figure 1:**
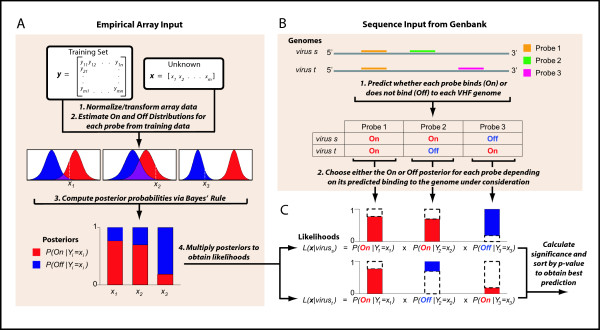
**Flow of VIPR**. The VIPR probabilistic model incorporates both empirical array data as well as sequence data from GenBank to calculate likelihoods for each candidate virus. A) Posterior probabilities are calculated for each probe. B) The *On *or *Off *posterior is chosen for each probe based on predicted binding to candidate genomes. C) Posteriors are multiplied to obtain a likelihood for each candidate virus.

For each sample in the training set, a unit-vector normalization was applied as shown, where *x_i _*represents the i^th ^intensity for a given hybridization. Then, each normalized intensity was log*_e _*transformed. As given in Equation (1), *x_i_^NT ^*is the normalized, transformed value for that intensity. Normalization was performed to account for variation in reagent concentrations or fluorescence across the microarray. Log transformation of the data was desirable for the estimation of normal distributions.(1)

Note that in the following calculations, all intensities have been normalized and transformed although the superscript *NT *does not appear.

### VIPR prediction of On and Off states (Figure 1B)

Candidate genomes to be scored in the VIPR algorithm were limited to all complete genomes in the NCBI virus RefSeq database as of 6/20/2008. The entire set of oligonucleotide probes on the microarray was aligned using BLASTN against each of the RefSeq viral genomes. Theoretical free energies of hybridization were then calculated from the aligned sequences using code included with OligoArraySelector [23]. If the free energy associated with binding of a given viral genome/oligonucleotide pair was computed to be less than -30 kcal/mol, the probe was assigned the *On *state for that genome; otherwise, the probe was assigned the *Off *state. The choice of -30 kcal/mol was based on the observation that this threshold represents the weakest binding reported for long-oligo broad specificity microarrays [23]. A given viral genome was included in the list of potentially detectable candidate viral genomes if at least three oligonucleotide probes were expected to bind to that genome (i.e. were expected to be *On*). A total of 101 candidate genomes met these criteria.

### VIPR calculation of posteriors (Figure 1A)

Posterior probabilities were calculated for each probe *i *according to Bayes' rule, (2) and (3). *Y_i _*and *x_i _*represent a random variable and an observed intensity, respectively.(2)(3)

Likelihoods for each probe were determined using normal distributions derived from two sets of normalized log*_e _*transformed intensities: those corresponding to the *On *states for a given probe (4), and those corresponding to the *Off *states (5).(4)(5)

The probe-specific *On *and *Off *distributions are derived from the training set where the probe *On*/*Off *states are defined by the identity of the virus in each hybridization.

### VIPR priors

Priors were calculated (6,7) in a probe-specific manner and were designed to incorporate two calculations derived from the composition of the microarray as well as the composition of the set of candidate viruses under evaluation: (a) the percentage of probes predicted to be *On *for the candidate virus under consideration, represented as *P(On)_pred_*; (b) the number of candidate viruses that share that probe's *On*/*Off *prediction (i.e. if four candidate viruses, including *virus_s_*, are predicted to be *On *for a given probe, then *P(virus_s_|On*) = 1/4 for that probe). Marginalizing over the possibility of an *On *or an *Off *prediction calls for a second invocation of Bayes' rule:(6)(7)

### VIPR calculation of hybridization likelihoods (Figure 1C)

Because of the possibility of underflow, all likelihood calculations were made in log space, though they are expressed here in probability space. The likelihood (8) of the observed hybridization vector, ***x***, was calculated for each of *n *viral genomes. The posteriors included in the product were chosen so as to reflect the expected state of a particular probe for *virus_s_*. *On *states for *virus_s _*are indexed from *i *= 1 to *a *while *Off *states are indexed from *j *= 1 to *b *as shown in formulas (9) and (10), respectively.

### Calculating significance of VIPR results

To determine the significance of the results obtained, we computed a p-value for each candidate virus by permuting the set of priors for the candidate over the set of likelihoods *P(Off *|*Y_i _*= *x_i _*) so as to estimate a null distribution of scores (n = 100 permutations) against which the actual score for that candidate could be compared. From the 100 null scores for each candidate virus, a mean and standard deviation were calculated. The resulting p-value reflects the percentage of the null distribution that is greater than or equal to the actual score. When assessing the significance of a given candidate, a Bonferroni correction was applied so that 0.05, a generally accepted level of significance, was divided by the total number of candidate viruses (101) i.e. a candidate was considered significant if its p-value was less than 5 × 10^-4^.

### Assessing the accuracy of VIPR

From the total 110 empirical hybridizations, 108 were chosen as suitable for training on the basis of percentage of well-behaved probes among those predicted to be *On*. Two hybridizations of Ippy virus to the array were excluded from training because the percentage of probes designed to bind to Ippy virus that evinced a sufficient separation (p < 0.001 by student's t-test) between the *On *and *Off *distributions was less than ten percent. For the initial cross-validation, the subset of 108 arrays was divided into a training set consisting of 107 arrays and a validation set consisting of a single array. This was done 108 times, leaving out a different array each time. The two arrays that did not meet the criterion for inclusion in the training set were tested using all 108 selected arrays for training. For each array, the best prediction was determined by sorting significant candidate viruses (p < 5 × 10^-4^) by p-value and then by likelihood. In the case where no virus was significant, the array was considered negative. Algorithm accuracy was computed using the formula, Accuracy = (TP + TN)/(TP + TN + FP + FN), where TP is the number of true positives, TN is the number of true negatives, FP is the number of false positives, and FN is the total number of false negatives. In the case where the fully sequenced genome of a viral subspecies was not available, an accurate prediction on the species level constituted a true positive. There was also one case where the genome of a subspecies (La Crosse virus) was used as a substitute for a hybridized species (California encephalitis virus) because the complete sequence of California encephalitis virus was not available. These designations of species and subspecies are according to NCBI taxonomy.

### Exploring alternative priors

In a separate analysis, VIPR's accuracy was assessed over a space of arbitrary priors rather than deliberately specifying priors using Equations (6) and (7). Thus, the marginalized priors *P(On)_marg _*and *P(Off)_marg _*in Equation (2) were replaced with priors that ranged iteratively from 0.1 to 0.9. For each iteration, one prior pair i.e. *P(On), P(Off*) where *P(Off) = 1- P(On*) was chosen for all *On *probes, while a separate pair was chosen for all *Off *probes. Thus, while the prior pair between the *On *and the *Off *probes could differ, the prior pair between any two *On *probes or between any two *Off *probes was the same. Hence, the space explored represents successive iterations of independently varying the *On *prior pair and the *Off *prior pair with variations made at a step size of 0.1. As before, p-values were calculated to assess the significance of VIPR results, except that 20 permutations were run for each candidate virus instead of 100.

### Exclusion of replicate hybridizations

Four independent strains of each of the following viruses, Crimean-Congo hemorrhagic fever, Guanarito virus and Omsk hemorrhagic fever virus were cultured in Vero cells. These viruses represent three of the four HF virus families. As with the other viruses in the positive control dataset, these viruses were hybridized in duplicate (3 viruses × 4 strains per virus × 2 hybridizations for each strain = 24 hybridizations). These 24 hybridizations were used to assess the effect that leaving out both replicates of a strain would have on cross-validation. VIPR predictions were made as described for the leave-one-out cross validation except that replicate hybridizations were excluded from training for the subset of 24 arrays. The number of accurate predictions made by VIPR out of the total 24 hybridizations was calculated.

### Comparison to existing diagnosis algorithms

Three algorithms, E-Predict, DetectiV and PhyloDetect, were available for comparison to VIPR. E-Predict [15] was used to calculate Uncentered Pearson correlations. A custom E-matrix for the HF dataset was prepared as described by Urisman et al. A given viral genome was included in the list of potentially detectable candidate viral genomes if at least three oligonucleotide probes were expected to bind to that genome. Default normalizations ('Sum' for the intensity vector and 'Quadratic' for the E-matrix) were applied. 110 correlations were used to estimate each null distribution of correlations from the set of HF arrays. These distributions were fit using the Shapiro-Wilk normality test as described [15]. The same significance threshold that was applied to VIPR predictions (p < 5 × 10^-4^) was also applied to E-Predict. Background-corrected intensities were loaded into DetectiV [16] and normalized in two independent ways: first using the median option, and second, against a Vero or C6/36 array serving as a negative control. The filtered results (mean log ratio > 1) for each array were then sorted by p-value to determine the top-scoring virus. The same significance threshold that was applied to VIPR predictions (p < 5 × 10^-4^) was also applied to DetectiV. Hybridization intensities were inputted to PhyloDetect [17] as binary vectors where a probe was considered 'present' if its intensity was greater than the median background signal plus twice the background standard deviation. The E-matrix constructed for E-Predict was converted to binary values (*x_i _*≥ -60 kcal/mol →1; otherwise → 0). The *fnr *parameter was set to 0.10. Results were sorted first by likelihood and then by number of present probes to determine the top candidate. A likelihood above the threshold 0.05 constituted a positive prediction. The same formula to calculate accuracy for VIPR was used to calculate accuracies for E-Predict, DetectiV and PhyloDetect.

## Results

RNA was purified from cell cultures that were infected with each of the viruses shown in Table 1. These viruses were selected to include almost all of the viruses known to cause HF; only a few very recently identified HF viruses, such as Chapare virus [24] and Lujo virus [25], were not included. To assess whether these viruses could be distinguished from close relatives that are not associated with HF, additional viruses were also selected from the same families for testing. For each of the 51 virus cultures, following random amplification and fluorescent labeling, two microarrays were hybridized generating a total of 102 empirical hybridizations using virally infected samples. In addition, eight negative control hybridizations (four from uninfected Vero cells and four from uninfected C6/36 cells) were performed.

We developed VIPR as an objective approach for analyzing diagnostic microarray data (VIPR is available for download from http://ibridgenetwork.org/wustl/vipr). VIPR incorporates both sequence data from GenBank as well as empirical array data to classify microarray hybridizations of samples with unknown viral infections (Figure 1). From these data, normal distributions corresponding to *On *and *Off *states for each probe were estimated.

**Figure 2:**
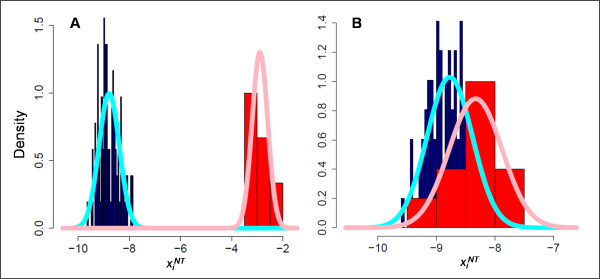
**Examples of *On *and *Off *distributions for two probes**. A) One representative probe with highly resolved *On *and *Off *distributions based on the training set data. B) One representative probe where the *On *and *Off *distributions overlap. Empirical distributions (blue = *Off*, red = *On*) and estimated distributions (cyan = *Off*, pink = *On*) are shown.

Empirical distributions and their normal approximations for two representative probes are shown in Figure 2. Figure 2A depicts a highly informative probe since there is effectively no overlap between the *On *and *Off *distributions for that probe. In contrast, the distributions in Figure 2B overlap substantially. Gradations between these two extremes constitute probes of intermediate informative value. Posterior probabilities were calculated via Bayes' rule for each probe given the observed intensity from an unclassified array. These posterior probabilities were multiplied to obtain likelihoods for each candidate virus [Additional file 1].

For identification of HF viruses, the algorithm was trained using a subset of the total 110 hybridizations. To select a suitable subset for training purposes, we identified 108 hybridizations for which at least 10% of the probes predicted to be *On *had intensities that differed significantly (p < 0.001) from that probe's *Off *distribution. To assess VIPR's performance on the 108 selected arrays, we performed leave-one-out cross validation so that the selected arrays were divided into a training set (n = 107) and a validation set (n = 1). The remaining two arrays (not included in the training set) were tested using the entire set of selected arrays (n = 108) for training.

An example of VIPR's output for a representative Dengue virus 3 hybridization is shown in Table 2. Likelihood scores for all candidate viruses for each microarray are available in the supporting material. We measured the accuracy with which we could make predictions for the virally infected and negative control arrays. VIPR made accurate predictions for 104 out the total 110 arrays. There were five false negatives and one false-positive, corresponding to an accuracy of 94%. The misclassified arrays are shown in Table 3.

**Table 2:** Five highest scoring candidates for a Dengue 3 hybridization.

Rank	Virus	Family	log(L)	p-value
1	Dengue virus 3	*Flaviviridae*	-352	0

2	Dengue virus 4	*Flaviviridae*	-391	0

3	Dengue virus 2	*Flaviviridae*	-539	0

4	Dengue virus 1	*Flaviviridae*	-599	0

5	Psittacid herpesvirus 1	*Bunyaviridae*	-433	1.0

**Table 3:** The six arrays that were misclassified by VIPR.

False positives		
Chip#	Hybridized virus	Top scoring virus (p < 5e-4)
207	Dengue virus 3	Dengue virus 4

**False negatives**		

Chip#	Hybridized virus	Top scoring virus (p < 5e-4)

462	Kyasanur Forest disease virus	none

463	Kyasanur Forest disease virus	none

464	Kyasanur Forest disease virus	none

221	Ippy virus	none

245	Ippy virus	none

**Figure 3:**
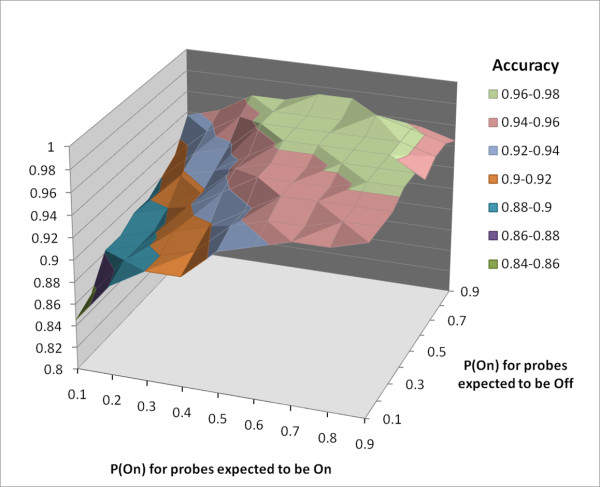
**Cross-validation results for different combinations of prior pairs**.

For all Bayesian methods, one question that must be addressed is how to choose appropriate priors. From many possible choices, we selected priors in this study based upon the composition of the probes in the microarray as well as the makeup of the candidate genomes under evaluation. In order to define the dependency of our algorithm's accuracy on the choice of priors, VIPR's accuracy was assessed over a range of possible prior pairs, independently varying the pair used for probes expected to be *On *versus the pair used for those expected to be *Off*. Hence, the space explored represents different combinations of prior pairs whose values lie between 0.1 and 0.9 with variations made at step size of 0.1, and with the sum of *P(On*) and *P(Off*) defined as 1.0 for each prior pair. 20 permutations were run for each candidate virus to compute p-values. Results are shown in Figure 3. Accuracy varied depending of the choice of priors, but remained fairly stable (between 85% and 97%) over a wide range of prior pairs, suggesting that the method is relatively insensitive to the choice of priors.

For the 24 hybridizations representing four strains from each of three species (Crimean-Congo hemorrhagic fever, Guanarito virus, Omsk hemorrhagic fever virus), a second cross-validation was performed in which both replicates corresponding to a particular strain were excluded from training when making VIPR predictions for those arrays. Leaving out both replicates for these particular strains was possible because there remained three other positive control strains of the same species in the training set. This could not be done in the case where only one strain of a species was present among the positive controls because it would render the training set devoid of any representatives of that species. VIPR analysis of the subset of arrays that represent viruses where multiple strains are present in the training set demonstrated robust prediction (24/24 arrays accurately predicted).

We compared the performance of VIPR to that of existing algorithms for analyzing diagnostic microarrays. E-Predict [15], the first algorithm expressly designed for interpretation of viral microarrays, uses a theoretical energy matrix to compute correlations between experimental hybridizations and genome-derived energy vectors [15]. As shown in Table 4, VIPR (94% accuracy) outperformed E-Predict (61% accuracy) for the same set of positive and negative control arrays. One possible explanation for E-Predict's low performance for this set of arrays is the lack of sufficient data to estimate accurate null distributions of scores by the Shapiro-Wilk criterion to be used to calculate p-values. For this dataset only 110 arrays were available for the estimation of null distributions for E-Predict, whereas over one thousand arrays were used by Urisman et al. [15] to calculate these distributions. This is supported by the fact that the virus with the highest raw score as determined by E-Predict is the true virus for 84 of the 102 positive control arrays.

**Table 4:** Accuracy of VIPR compared to other methods for this dataset.

Algorithm	Accuracy (%)
VIPR	94

DetectiV	76-83

E-Predict	61

PhyloDetect	49

DetectiV [16] is an R-based method for significance testing for microbial detection microarrays. Significance testing involves data normalization against one of the following: an array's median value for all probes, the mean value of a set of designated control probes, or a control array. No designated control probes, in the sense described by the DetectiV algorithm, were included in our design; therefore, the median and control array normalization options were used to analyze our data. After performing significance testing, the results were filtered to exclude groups whose mean log ratio was less than or equal to one. Sorting the filtered results by p-value then revealed a best prediction for each array. An accuracy of 69% was achieved using the median normalization method. Higher accuracies were achieved using the negative arrays with the control array normalization option. These accuracies ranged from 76% to 83% depending on which of the eight uninfected samples in our dataset was used as the control array.

PhyloDetect has previously been applied to viral diagnostic microarrays by increasing its 'false negative rate' parameter [17]. PhyloDetect, unlike VIPR, E-Predict and DetectiV, requires its hybridization inputs to be binary. To achieve this, we created a binary vector for each array where a probe was given a value of '1' if its intensity was greater than the median background signal plus twice the background standard deviation, and '0' otherwise. The theoretical microbial candidate profiles required for PhyloDetect are also binary. While the authors of PhyloDetect applied a stringent predicted binding energy threshold (-80 kcal/mol or less) to make binary present/absent predictions, our probe set, which included probes ranging in length from 35 to 60 nucleotides, could not tolerate such a stringent cutoff without resulting in some candidates having zero probes predicted as 'present.' Thus, we predicted a present probe when the corresponding binding energy was calculated to be -60 kcal/mol or less. After analysis of our data, we computed an accuracy of 49% for PhyloDetect.

## Discussion

The inherently parallel nature of DNA microarrays lends itself well to diagnostic applications seeking to simultaneously test for many microbial agents. While many diagnostic microarrays have been described [1-10] there is a relative lack of methods to objectively interpret these microarrays.

One key feature of a true diagnostic microarray is that the targets to be detected are typically well defined. Thus, specimens infected with these targets should be available for use as positive controls. In this study, we developed a novel interpretive algorithm for analysis of diagnostic microarrays that takes advantage of the existence of positive controls that can serve as a training set. VIPR performed with high accuracy (94%) as measured by leave-one-out cross validation. Since VIPR outperformed E-Predict, DetectiV and PhyloDetect for this dataset, this underscores the utility of using a set of known viruses together with a probabilistic algorithm to diagnose viral disease. Though we have not applied our algorithm to other diseases, we anticipate that this strategy would similarly be preferable to a non-Bayesian approach for diagnosis of other diseases of multiple etiologies whose microbial spectrum is well defined and for which positive and negative control specimens are available.

Only one false positive resulted from the cross-validation, which was a Dengue virus 3 sample being classified as Dengue virus 4. Dengue virus 3 was the second best prediction for this array, with both Dengue virus 4 and Dengue virus 3 achieving a p-value of 0.0. The other five microarrays that were misclassified by VIPR, all of which were called as virus negative, were derived from three virus cultures. None of these samples was accurately classified by E-Predict, DetectiV, or PhyloDetect. Given that these samples evaded accurate classification by all three algorithms, one possibility for the lack or detection of virus in these samples is the samples used as positive controls may have been present in abundance below the sensitivity limit of the microarrays. Another plausible explanation is that all or most of the probes designed to detect these viruses do not behave as predicted. In this case, redesigning the probes for these viruses would be the best way to improve the accuracy of the platform. Comparing the *On *and *Off *distributions for probes designed to bind to these viruses reveals that among those viruses that were hybridized to the array, Ippy virus and Kyasanur Forest disease virus exhibited the highest percentage of *On *probes that displayed no significant difference (p < 0.001) in intensity between the *On *and *Off *distributions (94% and 85% respectively). However, since VIPR's accuracy is inherently limited by the performance of the probe set, and the response of the probe set is determined by the identity and abundance of the target microbes, we are unable to distinguish between the possibilities of low-titer virus and misbehaving probes.

Other potential caveats related to our method include a limited ability to estimate the true intensity distribution of *On *states for a probe because of the small number of intensities in the training set that correspond to an *On *state. Hence, one way to improve the accuracy of estimation of these distributions would be to increase the number of positive control arrays in the training set. Depending on the degree of sequence divergence among the known strains of a given virus, it may also be important to represent the known diversity of related strains in the training set. However, we emphasize that even with the limited number of microarray hybridizations performed in this study, 94% accuracy was achieved.

The choice of prior probabilities could also be problematic in some circumstances. We found that prior estimation based on predicted binding of probes to viral genomes resulted in robust virus prediction. Moreover, accuracy remained fairly stable (between 85% and 97%) over a wide range of prior combinations. Another potential caveat with the VIPR algorithm is that the distribution of the log_e _of the intensities was assumed to be normally distributed. Gross violations of this assumption could have pejorative effects on prediction.

One limitation of a leave-one-out cross-validation in our case is that there is a possibility of overfitting due to the presence of replicate hybridizations in the training set. However, an analysis of a subset of arrays that represented several different strains of viruses (Crimean-Congo hemorrhagic fever virus, Guanarito virus and Omsk hemorrhagic fever virus) demonstrated that removing both replicate hybridizations for a given strain from the training set while retaining those from the other strains resulted in accurate prediction in every case. This subset of viruses represented three of the four families of HF viruses. While this analysis does not completely rule out the possibility of overfitting, it clearly demonstrates that VIPR can make accurate predictions even when replicate arrays are removed from training, as long as hybridizations representing strains from the same species are present. Additionally, VIPR outperformed the other three algorithms for this subset. E-Predict, DetectiV and PhyloDetect accurately classified 14, 16, and 8 of the 24 arrays, respectively.

While the results of our study represent a proof of principle using carefully controlled positive and negative controls for validation, it is anticipated that a probabilistic algorithm will be useful in clinical laboratory settings to analyze microarrays like the one described. Testing VIPR using clinical datasets will be the focus of future studies. In the case of diseases for which samples representing *in vivo *human infections are available, such would be the desired dataset for training. In the case of HF, however, clinical specimens from human infections are not generally available; therefore, it will be necessary to investigate the use of different kinds of specimens as training data for the probabilistic algorithm. These datasets could include specimens from infected animals or viruses harvested from culture and spiked into human sera.

As currently implemented, VIPR only looks for single virus effects. Possible improvements to the software might include the addition of functionality to detect the presence of co-infections and reassortant viruses. This could be accomplished by including among the list of candidates for which likelihoods are computed theoretical combinations of sets of *On *posteriors from different viruses. Equations (11) through (14) extend the single-virus likelihood calculation implemented by VIPR to the case where two viruses, *s *and *t *are present.(11)(12)(13)(14)

## Conclusions

We developed a probabilistic algorithm that relies on a training set of empirical hybridizations that accounts for probe-specific behaviors. Application of this algorithm to a dataset of cultured viruses that cause HF resulted in high accuracy virus identification. Though we report the application of VIPR only in the context of diagnosis of HF, our method of detection is theoretically applicable to any microbial detection problem in which a set of positive and negative control hybridizations is available. Our implementation of a probabilistic algorithm demonstrates the power of a Bayesian approach for discerning important hybridization signals from a complex mixture of nucleic acids. This, in turn, should prove to be of great value as microarray-based diagnostics play more prominent roles in clinical and public health laboratories.

## List of abbreviations

VIPR: refers to Viral Identification with a PRobabilistic algorithm; HF: refers to Hemorrhagic fever.

## Authors' contributions

AA developed and implemented the algorithm, performed all computational analyses and co-drafted the manuscript. GW and TW optimized and performed all nucleic acid preparations and microarray hybridizations. KF designed the microarray probes. MH and RT prepared virally infected cultures. DW designed the experiments, interpreted the data, conceived of and coordinated the study and co-drafted the manuscript. All authors read and provided feedback for the final version of the manuscript.

## Supplementary Material

Additional file 1 **VIPR output for all 110 HF hybridizations**. Click here for file
